# Bio-Resorption Control of Magnesium Alloy AZ31 Coated with High and Low Molecular Weight Polyethylene Oxide (PEO) Hydrogels

**DOI:** 10.3390/gels9100779

**Published:** 2023-09-25

**Authors:** Raffaella Aversa, Valeria Perrotta, Chao Wang, Antonio Apicella

**Affiliations:** 1 Advanced Materials Lab, Department of Architecture and Industrial Design, University of Campania, Via San Lorenzo, 81031 Aversa, Italy; raffaella.aversa@unicampania.it (R.A.); valeria.perrotta@unicampania.it (V.P.); 2 Key Laboratory of Biomechanics and Mechanobiology, Ministry of Education, Beijing Advanced Innovation Center for Biomedical Engineering, School of Biological Science and Medical Engineering, School of Engineering Medicine, Beihang University, No. 37, Xueyuan Road, Beijing 100083, China; 10896@buaa.edu.cn; 3 State Key Laboratory of Virtual Reality Technology and Systems, Beihang University, No. 37, Xueyuan Road, Beijing 100083, China

**Keywords:** bioresorbable implants, magnesium alloy, PEO, anomalous sorption, swelling, biocompatibility, corrosion

## Abstract

Magnesium AZ31 alloy has been chosen as bio-resorbable temporary prosthetic implants to investigate the degradation processes in a simulating body fluid (SBF) of the bare metal and the ones coated with low and high-molecular-weight PEO hydrogels. Hydrogel coatings are proposed to control the bioresorption rate of AZ31 alloy. The alloy was preliminary hydrothermally treated to form a magnesium hydroxide layer. 2 mm discs were used in bioresorption tests. Scanning electron microscopy was used to characterize the surface morphology of the hydrothermally treated and PEO-coated magnesium alloy surfaces. The variation of pH and the mass of Mg^2+^ ions present in the SBF corroding medium have been monitored for 15 days. Corrosion current densities (*I*_corr_) and corrosion potentials (*E*_corr_) were evaluated from potentiodynamic polarisation tests on the samples exposed to the SBF solution. Kinetics of cumulative Mg ions mass released in the corroding solution have been evaluated regarding cations diffusion and mass transport parameters. The initial corrosion rates for the H- and L-Mw PEO-coated specimens were similar (0.95 ± 0.12 and 1.82 ± 0.52 mg/cm^2^day, respectively) and almost 4 to 5 times slower than that of the uncoated system (6.08 mg/cm^2^day). Results showed that the highly swollen PEO hydrogel coatings may extend into the bulk solution, protecting the coated metal and efficiently controlling the degradation rate of magnesium alloys. These findings focus more research effort on investigating such systems as tunable bioresorbable prosthetic materials providing idoneous environments to support cells and bone tissue repair.

## 1. Introduction

In vivo and in vitro tests disclose that hydrogels represent a new type of functional polymeric material widely used in biomedicine because of their softness, high tissue water content, and good tissue compatibility. They may be applied in wound dressing, drug sustained-release carriers, oral protection, and scaffolding materials if they possess the required mechanical properties [1,2,3,4,5]. Although they are still developing, hydrogels have great potential for future clinical treatment. Several reviews have underlined the increasing development of new bone scaffolding materials with improved biocompatibility, non-immunogenicity, and toxicity, with a controlled degradable absorption and degradation rate that best match the formation of new bone acting as a bioactive interface between bone and metal rigid bone implants [6,7,8,9,10].

Rigid metals such as steel, titanium, and chrome-cobalt are widely used in prosthetic medicine applications due to their superior mechanical properties; however, local chronic inflammation, long-term endothelial dysfunction, retards in re-reendothelialization, thrombosis, and release of toxic metal ions are often observed and required secondary surgeries to remove them [10,11,12,13,14,15,16,17]. Biocompatible hydrogels, which are commonly utilized as not invasive scaffolding materials, have been described to potentially act as biomimetic and biomechanically active interfaces [18,19,20,21,22,23], presenting adequate porosities and structural properties to form three-dimensional structures with a large surface volume ratio able to favor osteogenic cells colonization [18,19,22].

Furthermore, advanced hydrogels may present multifunctional properties specifically designed to improve bone healing outcomes; enrichment with bioactive molecules, such as extracellular matrix proteins, adhesive peptides, growth factors, hormones, and silica or carbon-based nanoparticles, may be used to improve physicochemical as well as mechanical and biological properties of such polymeric scaffolding interfaces [18,19]. These structural hydrogels have been proposed as bio-mechanically active interfaces for implants’ early osteointegration [22]. The last few years have experienced significant advancements in hydrogel preparation by combining various organic and inorganic components to improve hydrogel physicochemical properties, enabling them to be cutting-edge biomaterials to translate into clinical applications [4,5,6].

Parallely, bioresorbable metal implants have been proposed to overcome long-term criticisms [6,7,8,9,10] of permanent metal implants. Among the candidate bioresorbable materials, magnesium attracted significant consideration for its lightweight, high mechanical specific properties, and compatibility with human physiology [8,9].

Magnesium-based implants possess osteoconductive properties experimentally testified by clinical trials where augmented peri-implant area bone apposition was observed [6]. Early studies [7,8,9,10] on bone fracture treatments have shown that Mg decreased the time for callous bone generation. The osteo-conductivity exhibited by magnesium has been suggested to derive from ion exchange with the biological environment [7,8,9]. Mg ions are a crucial co-factor for many enzymatic metabolic activities [6,8], facilitating tissue healing [9]. Moreover, they are rarely toxic and readily excreted [10].

Nevertheless, magnesium can’t be straightforwardly used as a structural material since it has extremely low intrinsic fracture toughness and high corrosion threats [10]. Magnesium alloys with improved mechanical and corrosion properties that do not contain high concentrations of metals that may induce long-term tissue damage [15,16,17,18,19,20], such as Ni, Al, Zr, and other rare earth metals, are then to be considered [11,12,13,14,15,16,17,18,19,20,21,22,23,24,25]. However, these alloys are described to embrittle and lose structural properties [14] due to magnesium’s low electrochemical potential fostering severe corrosion in chloride-containing environments like the human body [26,27]. Developing new technological solutions to reduce and control their degradation rates will favor the broader utilization of biodegradable temporary implants [22].

Corrosion rate control is mandatory to avoid excessive bio-resorption and loss of mechanical strength before tissue healing is accomplished [5]. Several techniques have been proposed to reduce and control the corrosion kinetics [14,22,23,24,25,26,27,28,29]. Among these, simple alkali heat treatment and polymer coatings [23,30,31,32,33,34,35,36,37,38,39] or more complex procedures such as Ion implantation [27,28,29] or micro-arc oxidation [34,35,36] can be adopted. Biodegradable polymer coatings represent the more accessible and well-performing technology [40,41,42,43,44,45,46,47].

The major weakness of using magnesium alloy in structural biomedical applications is its low corrosion resistance in electrolytic environments. This characteristic, if controlled, can be an intriguing property to developing bioresorbable orthopedic implants. Moreover, released magnesium ions play a stimulatory role in the growth of new bone tissue [27,28,29,30].

In our previous study, we chose the magnesium alloy AZ31 as a suitable bioresorbable bone implant material for its low contents of Al (3%) and Zn (1%) and good biocompatibility [11,12] and Poly-β-hydroxybutyrate (PHB) as a biocompatible and biodegradable [32] coating that successfully shield and controlled magnesium alloy corrosion rate [23]. However, although already used in several biomedical applications, tissue reaction to PHB degradation by-products is still wondered [42,43]. PHB and its copolymers naturally biodegrade in carbon dioxide and water by the human metabolism. Typical subcutaneous tissue reactions related to an increase in collagen deposition were reported in rats after implantation of PHB and attributed to mononuclear giant cell infiltration [43].

Bone remodeling processes repair the damage by removing and replacing the damaged tissues with new bone; this healing process can be strongly favored by using biocompatible and biomechanically active hydrogels that can be “designed” to reproduce bone-compatible and biomimetic structural interface with the rigid implants.

Due to their mechanical, physiological, and biomimetic characteristics, hydrogels can be good alternative candidates to control corrosion while supporting cells and tissues during bone repair, survival, proliferation, and differentiation, providing an idoneous microenvironment and extracellular matrix [48,49,50,51,52,53].

Coatings based on hydrophilic polymeric and ceramic-polymeric hybrid gels have been suggested for their tunable properties and biocompatibility advantages [39,48,49,50,51,52,53,54,55,56]. These synthetic polymeric hydrogels have been successfully adopted as tissue substitutes and drug delivery vehicles [39,44,45,46,47,48,49] with improved cytocompatibility and osteoindictivity [52]. Moreover, adding functional nanoparticles in polymeric hydrogels has further enhanced their mechanical and biological properties, overwhelming their often-criticized mechanical deficiencies [49,52,55]. In vitro experiments supported the hypothesis of the formation of hybrid ceramic-polymeric hybrids mating silica nanoparticles and acrylic hydrogels with significantly improved mechanical properties [39] and cell adhesion for murine fibroblasts and primary cultures of human OB [52]. In vivo, tests have shown a biomechanical activity of these hybrid coatings that favors the ability of OB to migrate from cancellous bone and adhere and colonize the peri-implant tissues [51].

Stable hydrogels are formed by polyethylene oxide (PEO) in the presence of aqueous media. When tested in the human body, PEO creates a stable to slightly soluble in the body fluids hydrogel that allows calcification inside the hydrogel, favoring the bone-bonding properties of the polymer [54]. Although PEO is water-soluble, swelling and dissolution depend on water activity and molecular weight [49,55]. Moreover, due to its hydrodynamic behavior [57,58,59], PEO hydrogel coatings reduce tissue adverse reactions to some biomaterials by shielding their intrinsic thrombogenicity [44,47]. These highly swollen water-soluble polymers may extend into the bulk solution [49], protecting the coated implant from plasma protein adsorption and avoiding platelet adhesion primarily by steric repulsion [45,46,47].

PEO hydrogel coatings could reduce the adhesion of microbial agents [45], resulting in promising coatings to prevent infection on medical implant surfaces. Their effectiveness, however, could be impaired by hydrolysis of the adhesive interface of the PEO chains to the implant surface as the morphological changes observed for swelling experiments in SBF [60].

Finally, the hydrogel interactions with body aqueous environments can affect structural and chemo-physical materials properties. Due to the different origins, functions, and clinical pathologies, physiological water may present different compositions and properties that need to be investigated to understand any possible hydrogel material properties modification.

The present work will evaluate the corrosion of a magnesium alloy (AZ31) exposed to SBF when coated with polyethylene oxide (PEO) of different molecular weights and when only hydrothermally treated to form a protective magnesium hydroxide layer. Our study will assess the protective coating potentials of a high molecular weight PEO (Mw = 4,000,000) since it is reported to swell to a greater extent and to form structural hydrogels able to reduce corrosion [54,55,56]. A PEO of lower molecular weight (Mw = 600,000) has been tested for comparison.

## 2. Results and Discussion

### 2.1. Morphological Characterisation of the Specimen’s Surfaces

The scanning electron microscopy (SEM) images (150 to 250×) of the surface of the uncoated hydrothermally treated and PEOs-coated specimens are reported in Figure 1A–C, respectively. The hydrothermally treated AZ31 magnesium alloy exhibits a continuous surface with visible micron grains of the magnesium hydroxide. Conversely, PEO-coated specimens are characterized by a homogeneous coating covering and adhering to the magnesium hydroxide [53].

**Figure 1:**
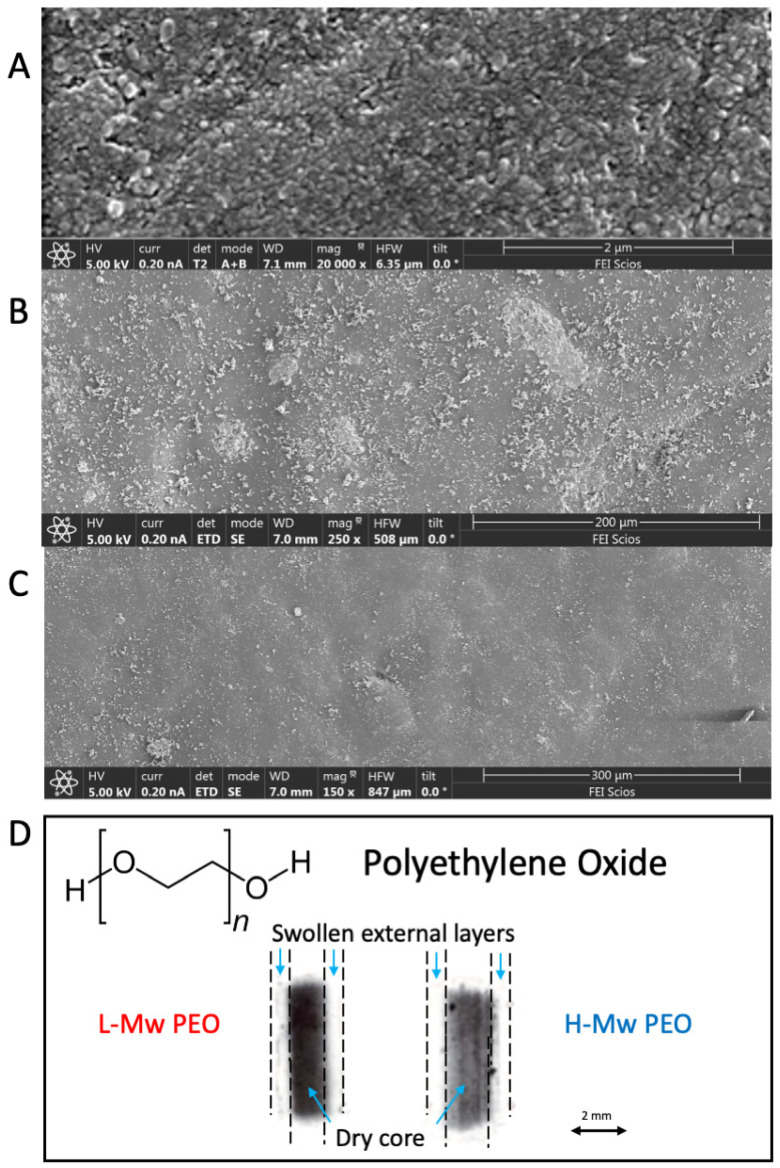
Electronic and optical microscopy characterization of the hydrothermally treated AZ31 and PEOs coatings surfaces. (**A**) AZ31 hydrothermally treated, (**B**) 600,000 Mw PEO, (**C**) 1,000,000 Mw PEO, and (**D**) micrograph of PEO anomalous sorption behavior in pure water (authorized elaboration from [49]).

Figure 1D shows a micrograft of the anomalous sorption experimentally observed [49] at 37 °C for L-Mw and H-Mw PEO tablets immersed in distilled water. Polyethylene oxide (PEO) is a nonionic polymer with good water affinity undergoing anomalous sorption, swelling, and dissolution in pure water. Figure 1D shows this anomalous sorption mechanism associated with swelling and relaxation of the initially dry polymer [49]: a sharp front divides the dry, unpenetrated core from the external swollen layer (Figure 1D). The swollen fronts move at a constant rate in the unpenetrated core. The authors have widely described this anomalous sorption phenomenon, identified as Case II, and how it can be used in control release applications for glassy and semicrystalline polymers with high affinity with the penetrant molecules [39,49,57,58].

Hydrogel water uptakes and solubilities are described to be significantly affected by external water activity [61] and polymer molecular weight [49]. From PEO molecular dynamic studies of flocculation processes [47], due to the molecular helicoidal conformation of poly(ethylene oxide) macromolecules with the ether oxygens outside, they are expected to behave differently than in pure water when in the presence of blood plasma containing several metal ions (see Table 4 in Section 4.1). Moreover, PEO swelling is strongly dependent on pH, higher in acid (pH < 7.4) than in basic (pH > 7.4) environments [56] and lower than those observed in the presence of pure water. As indicated in Figure 1D, these highly swollen water-soluble polymers may extend into the bulk solution [49], shielding the coated metal implant from corrosion predominantly by a steric ions repulsion mechanism [45,46,47].

### 2.2. Potentiodynamic Polarisation Tests

The polarization curves can obtain insights into the corrosion rate, passivity, pitting susceptibility, and the electrochemical system’s cathodic and anodic behavior. Natural corrosion current *I*_corr_ and corrosion potential *E*_corr_ intensities determine the type and the rates of anodic and cathodic electrochemical processes at the metal/corroding solution interface.

The larger the corrosion current density, the higher the corrosion rate and, hence, the lower the corrosion resistance of the metal [53,54].

**Figure 2:**
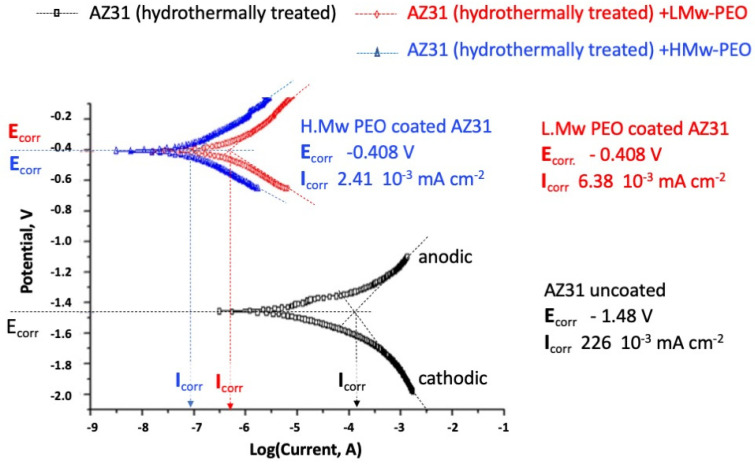
Potentiodynamic polarization curves in SBF solution held at 37 °C of bare hydrothermally treated (black squares) and the L-Mw (red circles) and H-Mw (blue triangles) PEOs coated AZ31 specimens.

The corrosion resistance tests of bare and PEOs coated AZ31 alloy specimens in the presence of SBF were conducted by potentiodynamic polarisation tests. Polarization curves of the AZ31 and the PEOs coated AZ31B disks in SBF solution are displayed in Figure 2. Corrosion current densities (*I*_corr_) and potentials (*E*_corr_) are determined from the intersection of the linear portions of the anodic and cathodic polarization curves reported in Figure 2

*E*_corr_ represents the corrosion potential where current density significantly increases up to its critical value according to the dissolution behavior of the metal.

The value of the corrosion current densities for the PEO-coated specimens are two to three folds lower than that of the uncoated AZ31 alloy. Because corrosion currents are proportional to the corrosion rate [38], a significant improvement in corrosion resistance is attained by both PEO coatings. The presence of the hydrogels could sterically hinder ion migration, reducing the reactions occurring at the metal interface. The H-Mw PEO behaves better than the L-Mw PEO among the two coatings.

The corrosion in the aqueous media of magnesium follows the reaction of water molecules with the metal-producing magnesium hydroxide and molecular hydrogen, implying the anodic reaction

Mg→Mg^2+^ + 2e^−^ (1)

and the cathodic reaction

2H_2_O + 2e^−^→H_2_ + 2OH (2)

**Figure 3:**
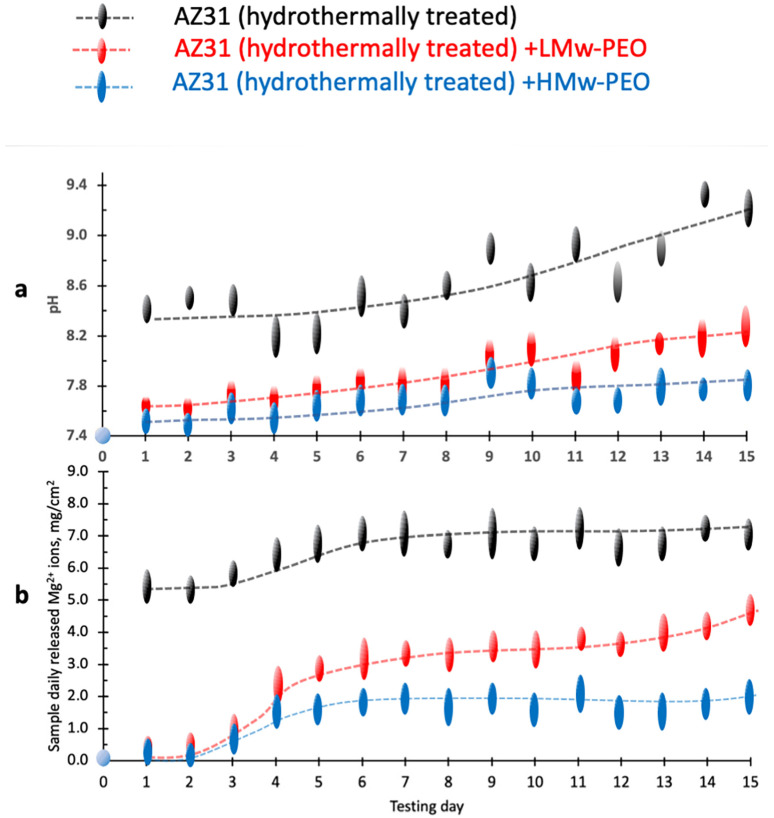
(**a**) pH of the SBF corroding medium, (**b**) mass of Mg^2+^ corrosion ions released in the SBF. The SBF corroding medium at 37 °C was refreshed daily.

The latter, (Equation (2)), determines an increase of the solution pH [53,54,55], as experimentally observed in our corrosion tests reported in Figure 3a.

After hydrothermal treatment, the alloy surface is covered by a magnesium oxide passivation layer. When this passivation layer is not protected during the polarisation tests, the metal cation transport controls the anodic Mg dissolution kinetic at the metal interface beneath the Mg(OH)_2_ passivation layer.

The porous structure of the Mg(OH)_2_ passivation layer does not efficiently hinder electrolyte mobility and diffusion in the aqueous medium. The cathodic reactions imply charge transfer freely occurring underneath and on the passivation hydroxide that can finally collapse, further increasing charge transfer and rapid advancement of the corrosion processes.

The uncoated alloy’s significantly higher natural corrosion current depends on the higher mobility charged species generated at the metallic interface by the failure of the passivation layer. For the PEO-coated alloy, conversely, the exchange current density from the cathodic reaction is reduced because the presence of the hydrogel acts as a physical barrier that retards water penetration and electrons and ions transport.

The anodic metal passivation and dissolution rate reduction occur as the PEO coatings significantly slow the diffusive mass transport of anions and cations [56].

Moreover, the formation of polar secondary PEO-Mg^2+^ bonds at the metal–polymer interface, which reflects the high number of sterically exposed Oxygens in the PEO hydrogel structure [47], may further hinder anodic reactions [56].

The layer of PEO coating is also retarding the diffusion in the bulk hydrogel of the anions from the SBF corroding medium to the metal interface resulting in a final natural corrosion current of about two to three folds lower than the bare AZ31 specimens.

The shift of the two PEO-coated alloy corrosion potential (*E*_corr_) towards the anodic direction of natural corrosion potential and the related passivation denote that the hydrogel is acting as a steric barrier that strongly hinders anions migration from the corroding solution to the cathode [56].

### 2.3. Wettability

Contact angle measurements investigated the surface wettability of AZ31 Mg alloy. The contact angles of water on the hydrothermally treated and HMw and LMw PEO-coated specimens are reported in Table 1.

The tests confirmed that the magnesium hydroxide layer of the hydrothermally treated specimens is homogeneous.

A mild hydrophilic water contact angle of 42.2° is shown by the hydrothermally treated AZ31 alloy. Conversely, the PEO polymer coatings increased the contact angles to 63.1° for the L-Mw PEO and 62.3 for the H-Mw PEO. These values are in the range of those reported in the literature [62,63].

The lower the hydrophilic nature of the PEO-coated external layer, the lower the surface wettability and the expected corrosion rate [52,53,54,55].

The surface wettability plays a relevant role in the corrosion and biological response of implanted devices because it affects the diffusion of water and solution electrolyte diffusion as well as the processes of corrosion and cell activity at the peri-implant area.

This could become particularly critical for magnesium alloy prostheses that, once whetted by the body fluids, activate corrosion and resorption processes that, if occurring at a high rate, are risky for their functionality [57].

The wettability of the hydrothermally treated magnesium specimens is higher (lower contact angle) than those of the PEO coatings. The lower wettability (higher contact angle) protects the metal surface, slowing erosion and lengthening implant functionality.

### 2.4. Simulating Body Fluid (SBF) Corrosion Tests

From the previous discussion, it can be inferred that an implanted magnesium prosthesis corrosion process and ions release in its surrounding biological environment are directly related to the electrochemical character of the reduction and oxidation reactions occurring between the metal interface and the external wetting electrolytic solution (plasma or SBF).

The results of 15-day SBF corrosion tests on L-Mw and H-Mw PEO-coated and uncoated alloy specimens are reported in Figure 3. The pH change of the SBF solution and the mass of the Mg^2+^ ions released have been recorded daily and are reported in Figure 3a,b, respectively.

Both PEO-coated Mg AZ31 specimens exhibited significantly different corrosion behavior in the curves compared to the uncoated system (Figure 3a). The pH values of the corroding medium for the H-Mw and L-Mw PEO-coated metal specimens rise from 7.4 to steady values ranging from 7.6 and 8.0, respectively (blue and red curves in Figure 3a).

In contrast, the uncoated Mg AZ31 specimens (black curve in Figure 3a) presented, since the first day of immersion in the corroding SBF solution, a subtle increase of pH denoting a higher basic behavior with pH values raising from 8.4–9.2 after 15 days of exposure.

For the uncoated samples, a second step rise of pH is observed on days 8–9. This event could be attributed to a collapse of the porous Mg(OH)_2_ [52,53]. The layer of porous hydroxide film, in fact, only moderately protects metal from the corroding SBF, only partially hindering and slower corrosion process [56,57]. Even if only marginally soluble in water, the magnesium hydroxide suffers from severe chemical modification in the presence of chloride ions. Reacting with Cl^−^ forms the highly soluble magnesium chloride and gaseous hydrogen [57], accompanied by a progressive increase of the external solution basicity and embrittlement of the Mg(OH)_2_ layer.

The increased pH observed on days 9–10 can be related to the collapse of the magnesium hydroxide layer and further metal exposure to the corroding medium.

The daily mass of Mg^2+^ released from the uncoated and PEO-coated magnesium is reported in Figure 3b as a function of the exposure day (from 1st to 15th). The daily mass released by the uncoated samples (black line in Figure 3b) is constantly higher than those registered for the L-Mw and H-Mw PEO-coated samples (red and blue lines, respectively)**.**

Consistently with the measurements of anodic and cathodic polarisation curves of Figure 2, a higher tendency to release Mg^2+^ ions should be expected for the bare alloy specimens vs. the hydrogel-coated ones; the natural corrosion currents of AZ31-coated PEOs are in fact, two folds lower than that of the bare alloy; namely 2.41 × 10^−^^3^ and 6.38 × 10^−3^ mA cm^−2^, respectively, for H-Mw and L-Mw PEO-coated vs. 2.26 × 10^−^^1^ mA cm^−2^ of the uncoated specimens. These electrochemical observations confirm the different corrosion processes hypothesized for bar and PEO hydrogel-coated AZ31 Mg alloy following immersion in the SBF. PEO coatings induce a higher diffusion resistance and lower cations and anions steric mobilities. It can be hypothesized that the degradation rate of the AZ31-coated PEO hydrogels could be significantly affected by the chemo-physical and diffusive characteristics of the coating film. In particular, acting as a membrane, it can steadily control the diffusive flux of the ionic species, principally Cl^−^ anions from the SBF solution to the metal surface and the counter-diffusion of Mg^2+^ ions toward the external solution. The hydrothermally induced magnesium hydroxide, less stable and brittle in the presence of chloride ions, does not similarly warrant a stable and efficient corrosion control.

**Figure 4:**
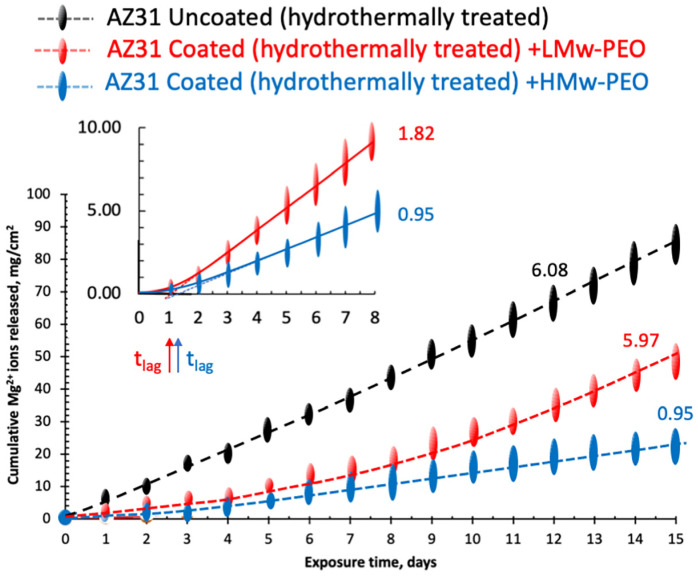
Cumulative Mg^2+^ Ions mass losses for bare thermally treated AZ31 Mg alloy and L-Mw and H-Mw PEO coated specimens. In the window: details of the initial part of the release curves with the evaluation of Mg ions time lag.

The cumulative amounts of magnesium ions daily released in the SBF solution (data taken from Figure 3b) from the bare and PEO hydrogel-coated AZ31 alloy are plotted as a function of the testing time in Figure 4.

A linear, steady, constant flux of Mg ions has been observed since early testing times (black line in Figure 4) for the bare alloy. The slope of this curve is reported in the diagram and is a measure of the corrosion rate that, in our case, is 6.08 ± 0.4 mg/cm^2^day.

A more complex behavior is observed in the early stages of the corrosion tests for the PEO-coated samples, and it needs a more detailed interpretation. Magnification of the initial portion of their cumulative Mg ions loss is reported in the left upper window of Figure 4.

An initial upward shape can be observed for both curves before attaining a steady constant slope. This transient behavior is characteristically observed in membrane permeability experiments. It is associated with the initial saturation of the polymeric interface followed by the diffusion of the sorbed species in the still unsaturated membrane core [49].

In the presence of the PEO hydrogels acting as membranes, a diffusive control is exerted on the mobility and permeation of the electrolytes present in the external corroding solution and those generated at the metal interface. This diffusive resistance is evident in a retard in reaching steady-state permeation (time lag). Time lags in our tests have been extracted from the intercept on the time scale (*t_lag_*) of the curves’ steady linear part: 1.11 ± 0.10 and 1.32 ± 0.05 days for L-Mw and H-Mw PEOs, respectively.

However, constant Mg ions release over the entire corrosion test is observed only for the H-Mw PEO-coated specimens. The corrosion rate evaluated from the steady slope of the the H-Mw PEO curve is reported in Figure 4, and it is almost four times lower than that of the uncoated system, namely, 0.95 and 6.08 mg/cm^2^day, respectively.

In contrast, after the time lag and an initial linear portion of the curve, a progressive increase in ion release rate is observed for L-Mw specimens (red line in Figure 4). The initial corrosion rate evaluated for L-Mw PEO-coated is 1.82 ± 0.52, higher than that of the H-Mw PEO. After the 11th day, the Mg^2+^ ions release rate reaches values comparable to that of the uncoated metal disks (not statistically different values of 5.97 ± 0.7 and 6.08 ± 0.4 mg/cm^2^day reported on the corresponding curves in Figure 4).

Permeability tests [58,59] characterize the permeation and diffusion coefficients of ions and molecules in polymers. The different concentrations of magnesium ions steadily generated by the corrosion reactions at the metal interface and that of the sane ions in the external corroding solution (daily refreshed) are the driving force for material flux.

Table 2 reports the swelling and diffusion parameters of L-Mw and H-Mw PEOs measured in the swelling and corrosion tests in the SBF aqueous medium.

Steady swelling ratios of 277.6 ± 18% and 260.5 ± 12% (the initial dry thickness) were reached after 0.54 h and 1.46 h for L-Mw and H-Mw PEOs, corresponding to 468 ± 20 and 180 ± 13 μm/h, respectively. SBF solution swells but does not dissolve H-Mw PEO film [64,65,66,67,68]; however, it could partially dissolve longer than the L-Mw PEO. The increased pH and Mg^2+^ ions release rate observed after 7–8 days of exposure to SBF in the L-Mw coated PEO could be attributed to a progressive dissolution of the protective coating (see Figure 4).

In our cumulative Mg ions mass loss curves, the diffusive resistance for their transport got from experimental transient time lags observed for the PEOs coated specimens. The diffusion coefficients of Mg^+2^ ions are evaluated using the standard time lag equation [58,59]:

D_Mg_^2+^ = *l*^2^/6 *t_lag_* (3)

where the *t_lag_* is the time lag (* 1.1 ± 0.1 and * 1.3 ± 0.2 days), *l* is the swollen coating thickness expressed in cm (* 0.099 ± 0.1 cm and * 0.094 ± 0.1 cm), and D_Mg_^2+^ is the resulting diffusion coefficients, which in our testing condition are * 1.70 ± 0.5 × 10^−8^ and * 1.32 ± 0.3 × 10^−8^ cm^2^/s for L-Mw and H-Mw PEO, respectively. Mg^2+^ ions diffusion coefficients, which account for the mutual cation and anion self-diffusion coefficients *D*+ and *D*− and solubilities in the polymer, are lower than those expected and observed for hydrophilic polymers (generally of the order of 10^−6^ cm^2^/s) [64]. Due to the polar nature of the hydrogel matrix, a dual-mode sorption behavior can be hypothesized [65,66,67]. The Mg^2+^ cations diffusion in the PEO bulk coating toward the external SBF solution is slowed down by the temporary immobilization of the matrix polar groups [47]. It also occurs to the counter diffusion of the Cl^−^ ions directly involved in the metal corrosion process. The polymer film, therefore, acts as a physical barrier that sterically hinders electrolyte anions migration from the external SBF corroding solution to the metal [68,69,70].

## 3. Conclusions

The PEO dip-coating method is a simple process for surgical bioresorbable implants to improve and control their bio-corrosion rate.

The polymer coating, coupled with hydrothermal treatment, forms a magnesium hydroxide film on the alloy metal surface, favoring hydrogen bonding with the PEO oxygen and improving the adhesion between the polymer coating and the magnesium surface. The potentiometric data demonstrates that the hydrogel polymeric coatings sterically hinder the transport of Mg cations from the metal substrate. The hydrogel film acts as a membrane that steadily controls the diffusion of Cl^−^ ions from the SBF solution to the metal surface and the counter-diffusion of released Mg^2+^ metal ions toward the surrounding solution. SBF corrosion test showed that under the same experimental conditions, the loss rate of magnesium ions is significantly reduced by the presence of the polymer hydrogel coating. The initial corrosion rates for the H- and L-Mw PEO-coated Mg alloy are similar (0.95 ± 0.12 and 1.82 ± 0.52 mg/cm^2^day, respectively) and almost four to five times slower than that of the uncoated system (6.08 mg/cm^2^day), respectively.

Polymeric and hydrogel coatings, while enhancing the corrosion resistance of magnesium alloy, can also be used according to their diffusive characteristics to design targeted bio-resorption times. The control of the resorption rates after implantation of and, hence, of the Mg^2+^ ions concentration in the biological fluids could broaden the application areas of these systems. Magnesium and its alloys could be used as bioresorbable lightweight, structural orthopedic implants, maintaining mechanical integrity over a planned time scale before complete resorption is replaced by natural tissue [31,32].

In the limits of a single specific simulating body fluid (SBF), the use of only two limiting PEOs molecular weights and the absence of a viscoelastic mechanical characterization of the SBF swollen polymers, this preliminary study demonstrates that the PEO hydrogel coating can effectively avoid, in a potential clinical application of this Mg alloy, the detrimental effect of an intense and uncontrolled release in the body of Mg ions by reducing the metal degradation rate. Further clinical in vivo testing is then needed to confirm these expectations.

## 4. Materials and Methods

### 4.1. Materials

Magnesium AZ31 12 mm bars (Auremo GmbH, Saarbrücken Germany) with the chemical composition reported in Table 3 were used to prepare the bioresorbable metal disc specimens. Reagent grade polyethene oxide (PEO) of average Mw’s 600,000 and 4,000,000 (Sigma-Aldrich catalog 182,028 and 189,464, respectively) were used to coat the Mg alloy disks.

Simulated body fluids solutions mimicking blood plasma containing the ionic species reported in Table 4 have been prepared according to standard accepted procedures [71], resulting in an aqueous medium with a water activity of 0.975 (determined by vapor pressure measurements) and pH of 7.4.

### 4.2. Preparation of Mg Alloy Disks and PEO Films and Coatings on Metal Disks

#### 4.2.1. Magnesium Alloy AZ31 Specimens

Disks with dimensions of 2 mm thickness were cut from a 12 mm bar of AZ31 magnesium alloy mechanically ground with 1200 SiC paper. Before undergoing coating procedures, the specimens were ultrasonically cleaned in acetone, rinsed in ethanol and distilled water, and hydrothermally treated for 2 h at 100 °C to obtain a uniform and homogeneous Mg(OH)_2_ sample surface with improved anticorrosion and adhesion properties [52].

#### 4.2.2. Preparation of PEO Coating Solutions and Film and Coating Deposition

L-Mw and high Mw PEO were dissolved in analytical grade chloroform (Sigma-Aldrich-Italia s.r.l., Milano, Italy) at a concentration of 5 wt%. Dip coating of the AZ31 alloy disks in the PEOs solutions and solution film casting on glass support were then carried out, followed by solvent evaporation at 20 °C for 24 h. The PEO films (detached from the glass support) and the polymer-coated and hydrothermal-treated control AZ31 alloy specimens were stored in a dry environment.

#### 4.2.3. PEOs Film and Coating Thickness Measurements

The thicknesses of the dry and equilibrated SBF solution cast films were measured with an optical stereo scan microscope (Leica EZ4 W). The dry cast films of L-Mw and H-Mw PEO films were equilibrated in the SBF solution until constant swollen thickness was reached. Swelling rates (μm/h) and ratios (%) for the two polymers were evaluated from the times needed to get a steady thickness *t_eq_* and from its final value *l_sw_* according to:

Swelling rate = (*l_sw_* − *l_dry_)/t_eq_* (4)

and,

% Swelling ratio = 100 *l_sw_/l_dry_* (5)

An indirect measure of the disk’s polymeric coatings thickness was gravimetrically determined. The specimen weight increase after the coating and drying procedures was attributed to the forming of a thin polymer film. Considering a uniform distribution of the film on the surface area of the metal disk (3.47 cm^2^), and for both polymers, a density of 1.21 g/cm^3^ (Sigma-Aldrich product specifications), the gravimetric calculated mean coating thicknesses were 252 ± 16 μm and 246 ± 11 μm for L-Mw and H-Mw PEO, respectively.

### 4.3. Surface Morphological Characterisation

The surface morphologies of the hydrothermally treated specimens before and after PEOs coating were analyzed by scanning electron microscopy (FEI Scios™ Hillsboro, OR, USA).

### 4.4. Electrochemical Analysis

Electrochemical measurements were made utilizing a Model 600 series electrochemical analyzer workstation (CH Instrument Inc., Austin, TX, USA) with three electrodes: the hydrothermally treated and PEOs coated specimens were used as the working electrode (1 cm^2^ of effective exposed area), platinum as a counter-electrode and a saturated calomel reference electrode. A 50 mL deoxygenated SBF thermostat at 37 ± 0.2 °C served as an electrolyte. Scanning 2 mV/s from −2 V to −1 V obtained potentiodynamic polarisation curves.

The natural corrosion current (*I*_corr_) and corrosion potential (*E*_corr_) were determined by extrapolating the intercepts of the anodic and cathodic lines at the zero current potential.

### 4.5. AZ31 Alloy Degradation Tests

The metal corrosion tests in SBF of the reference uncoated and PEOs-coated magnesium AZ31 alloy specimens were conducted at 37 ± 0.2 °C. The *solution volume/sample area* ratio was 30 mL/cm^2^ (the standard requirement is a value of 20 mL/cm^2^ to 40 mL/cm^2^). Compositions of the SBF used in our corrosion test and human blood plasma are compared in Table 4.

The coated and reference specimens were immersed in flasks with SBF solution. Equal volumes of corroding solution were collected daily to measure their pH and the concentration of magnesium ions detected by the inductively coupled Agilent 7850 ICP plasma mass spectrometer. After each sampling, SBF solutions were renewed daily during the 15 days of corrosion tests.

Inductively coupled plasma mass spectrometry evaluated corrosion rates as daily released Mg^2+^ ions mass (mg/cm^2^ per day).

### 4.6. Statistical Analysis

Four measures for each experiment were taken, and the data were expressed as the mean and the standard deviation (Mn ± SD).

Comparisons among values for all groups were performed by one-way analysis of variance (ANOVA). Differences were considered significant (*) for *p* < 0.05 and highly significant (**) for *p* < 0.01.

## Figures and Tables

**Table 1:** Water contact angles for AZ31 Mg alloy hydrothermally treated with the formation of a Mg(OH)_2_ (first column), hydrothermally treated + L-Mw PEO surface coating (second column), and hydrothermally treated + H-Mw PEO coating (third column).

AZ31 Mg Alloy + Mg(OH)_2_	AZ31 Mg Alloy + Mg(OH)_2_ + L-Mw PEO	AZ31 Mg Alloy + Mg(OH)_2_ + H-Mw PEO
* 42.2° ± 0.4°	* 63.1° ± 0.4°	** 62.3° ± 0.3°

* significant difference (*p* < 0.05), ** highly significant difference (*p* < 0.01).

**Table 2:** L-Mw PEO and H-Mw PEO coatings swelling and corrosion protection parameters.

PEO	Swelling Rate, μm/h	Swelling Ratio, %	Dry Thickness ^1^, μm	^2^ Hydrogel Thickness, μm	*t_lag_*, Days	D_Mg+2_ cm^2^/s	Corrosion Rate, mg/cm^2^day
L-Mw 600,000	* 468 ± 20	277.6 ± 18	* 252 ± 16	* 999.7	* 1.11 ± 0.10	1.70 ± 0.5 × 10^−8^	* 1.82 ± 0.52
H-Mw 4,000,000	** 180 ± 13	260.5 ± 12	* 246 ± 11	* 942.0	* 1.32 ± 0.05	1.32 ± 0.3 × 10^−8^	* 0.95 ± 0.12

^1^ measured by gravimetric method, ^2^ optically measured after swelling test. * significant difference (*p* < 0.05), ** highly significant difference (*p* < 0.01).

**Table 3:** Chemical composition of the as-received AZ31 Magnesium bar.

Metal	Wt %
Mg	96.12
Al	2.89
Zn	0.92
Mn	0.05
Si	0.01
Cu + Ni + Fe	0.01

**Table 4:** The concentration of inorganic ions in blood plasma and SBF.

Ions	Plasma, mmol/L	SBF, mmol/L
Na^+^	142.0	142.0
K^+^	5.0	5.0
Mg^2+^	1.5	1.5
Ca^2+^	2.5	2.5
Cl^−^	103.0	147.8
(HCO_3_)^−^	27.0	4.2
(HPO_4_)^2−^	1.0	1.0
(SO_4_)^2−^	0.5	0.5
pH	7.2–7.4	7.4

## Data Availability

Not applicable.
